# Antidiabetic Activity of *Aloe vera* Leaves

**DOI:** 10.1155/2020/6371201

**Published:** 2020-05-24

**Authors:** Alethia Muñiz-Ramirez, Rosa M. Perez, Efren Garcia, Fabiola E. Garcia

**Affiliations:** ^1^CONACYT-IPICYT/CIIDZA, Camino a la Presa de San José 2055, Col. Lomas 4 Sección, CP 78216 San Luis Potosí S.L.P, Mexico; ^2^Laboratorio de Investigación de Productos Naturales, Escuela Superior de Ingeniería Química e Industrias Extractivas, Instituto Politécnico Nacional, Av. Instituto Politécnico Nacional S/N, Unidad Profesional Adolfo Lopez Mateos, CP 07708 Ciudad de Mexico, Mexico; ^3^Laboratorio de Química Supramolecular y Nanociencias, Instituto Politécnico Nacional, Acueducto S/N, Barrio la laguna Ticomán, CP 07340 Ciudad de México, Mexico; ^4^Facultad de Ciencias Químicas, Universidad Autónoma de San Luis Potosí, Av. Dr. Manuel Nava No. 6-Zona Universitaria, CP 78210 San Luis Potosí S.L.P, Mexico

## Abstract

This research evaluated the potential of using the methanol extract of *Aloe vera* (L.) Burm.f (AVM) to prevent the formation of AGEs by means of the BSA/glucose assay, BSA-methylglyoxal assay, arginine-methylglyoxal assay, fructosamine, N*ɛ*-(carboxymethyl) lysine (CML), thiol groups, and carbonyl protein *in vitro*. The effect of AVM was also evaluated with regard to inhibiting the enzymes *α*-amylase, *α*-glucosidase, and pancreatic lipase. For this, the plant was dried, ground, and subsequently macerated with methanol. *Aloe vera* methanol extract (AVM) significantly decreased the formation of AGEs, as well as the formation of fructosamine, CML, and carbonyl protein. The concentration of 5 mg/ml of AVM presented the best results. AVM significantly inhibited the *α*-amylase and *α*-glucosidase enzymes. As regards the content of thiol groups, a significant increase was observed during the four weeks of the experiment. So, we can conclude that *Aloe vera* methanol extract decreases the formation of AGEs and may inhibit the increase in postprandial glucose, suggesting that AVM can prevent diabetes complications associated with AGE.

## 1. Introduction

Protein glycation is considered one of the main causes of complications of diabetes, such as vasculopathy, retinopathy, nephropathy and neuropathy, cataracts, and chronic kidney disease [1]. The glycation of proteins brings about the formation of advanced glycation end products (AGEs), which modify the structure of proteins and alter enzymatic activity [2]. AGEs are formed by the nonenzymatic reaction of the free amino group of a protein with the carbonyl of a sugar or a reducing aldehyde (called the Maillard reaction) [3], resulting in the formation of Schiff bases, subsequently through a series of reactions, and fluorescent compounds such as pentosidine and nonfluorescent compounds such as carboxymethyl lysine (CML) are formed [4]. Some AGEs, such as CML and pentosidine, have become biomarkers for glycooxidizing damage [5]. Therefore, taking into account the pathological implications of glycation, it becomes essential to discover inhibitors of protein glycation, which may help reduce and/or prevent complications in diabetes mellitus [2]. For centuries, herbal medicines have been widely used to treat a wide variety of diseases. To date, many of these plants are still used as a first alternative to cure certain diseases in developing countries around the world due to the few side effects they present; it has also been reported that around 20% of the medicines used throughout the world come from plants [6]. In this context, there is a growing interest in discovering safe and nontoxic plant sources to find alternative or complementary medicines that help the treatment of various chronic diseases such as diabetes mellitus. *Aloe vera* (L.) Burm.f. has been used by different cultures such as the Egyptian, Indian, Chinese, and European cultures for more than 5000 years due to its extraordinary medicinal properties [7, 8]. The genus *Aloe* grows in arid, tropical, and subtropical areas; this genus includes approximately 450 species. It is a succulent plant with no stem or a short stem and can grow to be 60−100 cm high; its leaves are fleshy, thick, triangular, and spiny [8], which gives the appearance of cactus, but in fact it belongs to the lilac (Liliacea) family. Its leaves have the ability to retain water, which allows the plant to survive in environments with long periods of drought, where most of the vegetation disappears [9]. It contains more than 70 active compounds [7], including vitamins, minerals, enzymes, polysaccharides, phenolic compounds, and organic acids. It has been reported that the polysaccharides present in the *A. vera* gel have therapeutic properties such as anti-inflammatory, healing, antibacterial, antioxidant, anticarcinogenic, antidiabetic, and antiaging properties, among others [8, 9]. Taking into account the pathological effects that glycation brings about, it is very important to find compounds that inhibit the nonenzymatic glycation of proteins to help prevent the generation of secondary complications, which is why this study's objective was to assess the capacity of the methanol extract of *Aloe vera* (AVM) to inhibit the protein glycation reaction by means of the BSA/glucose assay.

## 2. Materials and Methods

### 2.1. Collection of Raw Materials

*Aloe vera* was collected in May 2018, in the municipality of Armadillo, San Luis Potosí. A specimen was taken to the herbarium of the Autonomous Metropolitan University for future reference (specimen number ARC-53578).

### 2.2. Preparation of the Extract

Three hundred grams of the whole leaf was dried and pulverized in a mechanical mill, obtaining 110 g of dry weight. The powdered material was extracted with 3 L of methanol using a Soxhlet apparatus. The extract (AVM) was filtered and concentrated by a rotary vacuum evaporator for the complete removal of solvents.

### 2.3. Inhibition Tests for the Formation of Advanced Glycation End Products

#### 2.3.1. *In vitro* Glycation of Bovine Albumin

The glycation of the BSA was carried out using the method used by Gutierrez et al. [10] with some modifications. The AVM extract was dissolved in DMSO (0.031–0.500 mg/ml), adding 10 mg/ml of BSA, 1.1 M glucose, 0.1 M phosphate buffer at pH 7.4, and 0.2% sodium azide. The solution was incubated at 37°C for four weeks. The formation of the glycated BSA was determined at an excitation wavelength of 355 nm and emission of 460 nm; aminoguanidine was used as a positive control.

#### 2.3.2. Determination of Fructosamine

The AVM extract was dissolved in DMSO (0.031–0.500 mg/ml), adding 10 mg/ml of BSA, 1.1 M glucose, 0.1 M phosphate buffer at pH 7.4, and 0.2% sodium azide. The solution was incubated at 37°C for four weeks. After four weeks of incubation, the fructosamine concentration of the Amadori product was assessed by the nitroblue tetrazolium assay (NTB) [11]. 10 *μ*l of glycated BSA was incubated with 90 *μ*l of 0.5 mM NTB, in 0.1 M carbonate buffer at a pH of 10.4 at 37°C. After 10 to 15 minutes, the absorbance was read at 530 nm. The concentration of fructosamine was calculated by comparing it with 1-deoxy-1-morpholino-fructose (1-DMF), which was used as standard.

#### 2.3.3. BSA-Methylglyoxal Assay

This test was performed to evaluate the middle stage of protein glycation. Methylglyoxal was added (60 mM, 1 ml), at different concentrations of AVM (0.30, 0.60, 1.2, 2.5 and 5 mg/ml) and catechin (1.5 mg/ml, 1 ml), in sodium phosphate buffer (50 mM, pH 7.4) and sodium azide (0.02%) at 37 ° C for 2 h. BSA (30 mg/ml, 1 ml) was subsequently added to the mixture and incubated at 37° for six days. The phosphate buffer (1 ml) was used as a negative control without the addition of AVM extract. As a positive control, aminoguanidine (10 mM, final concentration) was used. After the incubation time, the samples were read in a microplate reader at 340 nm excitation and 380 nm emission [12]. The percentage of inhibition of AGEs was calculated using

(1) $$\begin{array}{c} \text{ percentage inhibition}=1-\left(\frac{\text{fluorescent intensity with inhibitor}}{\text{fluorescent intensity without inhibitor}}\right)\times 100. \end{array}$$

#### 2.3.4. Arginine-Methylglyoxal Assay

This test analyzes the main and specific source of AGEs formation. Methylglyoxal was added (60 mM, 1 ml), at different concentrations of AVM (0.30, 0.60, 1.2, 2.5, and 5 mg/ml) and catechin (1.5 mg/ml, 1 ml), in sodium phosphate buffer (50 mM, pH 7.4) and sodium azide (0.02%) at 37°C for 2 h. Subsequently, arginine (60 mM, 1 ml) was added to the different samples and incubated at 37°C for six days. As a negative control, phosphate buffer (1 ml) was used without the addition of AVM extract. As a positive control, aminoguanidine (10 mM, final concentration) was used. After the incubation time, the samples were read on a microplate reader at 340 nm excitation and 380 nm emission [12]. The percentage of inhibition of AGEs was calculated using (1).

#### 2.3.5. Determination of N*ɛ*-(Carboxymethyl) Lysine

At the end of the fourth week of incubation, the N*ε*-(carboxymethyl) lysine (CML) was determined, and a major antigenic AGE structure was determined by using the enzyme linked immunosorbant assay (ELISA) kit. The concentration of CML was calculated by using the standard CML-BSA curve from the assay kit.

### 2.4. Determination of Protein Carbonyl Content

After four weeks of incubation, the content of the carbonyl group was determined in glycosylated BSA, which is a marker of oxidative protein damage, using the method described by Joglekar et al. [2] with some modifications. 800 *μ*l of 10 mM 2-4-dinitrophenylhydrazine (DNPH) in 2.5 M HCL was added to 200 *μ*l of glycated samples. The samples were allowed to incubate in the dark for 1 h, and then the proteins were precipitated using 1 ml of trichloroacetic acid. (TCA) at 20% (p/V), leaving the samples on ice for five minutes, and they were then centrifuged at 10,000 g for 10 min at 4°C. The supernatant was washed three times using 500 *μ*l of a mixture of ethanol/ethyl acetate (1 : 1), and then the protein supernatant was dissolved in 500 *μ*l of 6 M guanidine hydrochloride. The absorbance reading was 370 nm. The carbonyl group concentration of the samples was calculated using the absorption coefficient (*ɛ* = 22,000M^−1^ *∗* cm^−1^). The results were expressed as nmol carbonyl/mg protein.

### 2.5. Thiol Group Estimation

After four weeks of incubation, free thiol groups were determined in glycated BSA using the method described by [11] with some modifications. 70 *μ*l of the sample was incubated with 130 *μ*l of 5 mM 5,5′-dithiobis (2-nitrobenzoic acid) (DTNB) in 0.1 M PBS, pH 7.4 at 25°C for 15 minutes. After the absorbance of the samples was read at 412 nm, the concentration of free thiol was calculated using a standard curve of L-cysteine. The results were expressed as nmol/mg of protein.

### 2.6. Enzymatic Assays

#### 2.6.1. Inhibition of *α*-Amylase

The inhibition of *α*-amylase activity was performed using a modified method with 2-chloro-4-nitrophenyl-4-*β*-D-galactopyranosylmaltoside (GalG2CNP) as substrate and saliva fraction enriched with *α*-amylase (HSA-f) [13]. The HSA-f was diluted 100-fold in 50 mmol L^−1^ 2-(N-morpholino) ethanesulfonic acid (MES) buffer containing 5 mmol L^−1^ CaCl_2_, 140 mmol L^−1^ potassium thiocyanate, and 300 mmol L^−1^ NaCl (pH 6.0). Different concentrations of AVM (0.30, 0.60, 1.2, 2.5, and 5 mg/ml) previously diluted in 5% DMSO were incubated with HSA-f (1 : 10 v v^−1^) at 37°C for 30 min. The reaction was initiated by adding 12 mmol L^−1^ GalG2CNP substrate; increases in absorbance (i.e., CNP release) were measured at 37°C at 405 nm. This assay was also carried out with acarbose (purity > 95%) as a positive control and 5% DMSO as a negative control. The results were presented as a percentage of *α*-amylase inhibition (AI), calculated according to the following:

(2) $$\begin{array}{c} \text{AI}\left(\%\right)=\frac{\left(\;A_{\text{nc}}-A_{\text{sample}}\right)}{A_{\text{nc}}}\times \;100, \end{array}$$

where *A*_nc_ is the absorbance of negative control and *A*_sample_ is the absorbance of the extract incubated with HSA-f.

#### 2.6.2. Inhibition of *α*-Glucosidase

The inhibition of *α*-glucosidase activity was performed using a modified method with 4-nitrophenyl *α*-D-glucopyranoside (p-NPG) as substrate and *α*-glucosidase-enriched fraction from intestinal acetone powders from rats (AG-f) [13]. Different concentrations of AVM (0.30, 0.60, 1.2, 2.5, and 5 mg/ml), previously diluted in 5% DMSO, were incubated with AG-f and 1.5 mmol L^−1^ reduced glutathione (diluted in 50 mmol L^−1^ phosphate buffer, pH 6.8) for 20 min at 37°C. The reaction was started by adding 4 mmol L^−1^ p-NPG (diluted in 50 mmol L^−1^ phosphate buffer, pH 6.8) and absorbance values were measured at 405 nm for 30 min at 37°C. This assay was also carried out with acarbose (purity > 95%) as a positive control and 5% DMSO as a negative control. The results were presented as a percentage of *α*-glucosidase inhibition (GsI), calculated according to the following:

(3) $$\begin{array}{c} \text{GsI}\left(\%\right)=\frac{\left(\;A_{\text{nc}}-A_{\text{sample}}\right)}{A_{\text{nc}}}\times \;100, \end{array}$$

where *A*_nc_ is the absorbance of negative control and *A*_sample_ is the absorbance of the extract incubated with AG-f.

#### 2.6.3. Inhibition of Pancreatic Lipase

The inhibition of pancreatic lipase activity was performed using a modified method with p-nitrophenyl palmitate (p-NPP) as substrate and porcine pancreatic lipase (PL) (type II, Sigma) [14]. Different concentrations of AVM (0.30, 0.60, 1.2, 2.5, and 5 mg/ml), previously diluted in 5% DMSO, were incubated with 10 g L^−1^ PL (diluted in 50 mM Tris-HCl buffer pH 8.0, containing 10 mmol L^−1^ CaCl_2_ and 25 mmol L^−1^ NaCl) for 20 min at 37°C. The reaction was started by adding 0.8 mmol L^−1^ p-NPP substrate (diluted in 10% isopropanol and 50 mmol L^−1^ Tris-HCl buffer, containing 10 mmol L^−1^ CaCl_2_, 25 mmol L^−1^ NaCl, and 0.5% Triton X-100), and absorbance values were measured at 410 nm for 30 min at 30°C. This assay was also carried out with orlistat (purity > 98%) as a positive control and 5% DMSO as a negative control. The results were presented as a percentage of lipase inhibition (LI), calculated according to the following:

(4) $$\begin{array}{c} \text{LI}\left(\%\right)=\frac{\left(\;A_{\text{nc}}-A_{\text{sample}}\right)}{A_{\text{nc}}}\times 100, \end{array}$$

where *A*_nc_ is the absorbance of negative control and *A*_sample_ is the absorbance of the extract incubated with PL.

### 2.7. Statistical Analysis

The results were expressed as the mean ± standard error of the mean (SEM) (*n* = 3). The statistical significance of the results was evaluated by using one-way ANOVA. The least significant difference (LSD) test was used for mean comparisons, and *P* < 0.05 was statistically significant.

## 3. Results

### 3.1. Inhibition Tests for the Formation of Advanced Glycation End Products

#### 3.1.1. *In vitro* Glycation of Bovine Albumin

**Figure 1:**
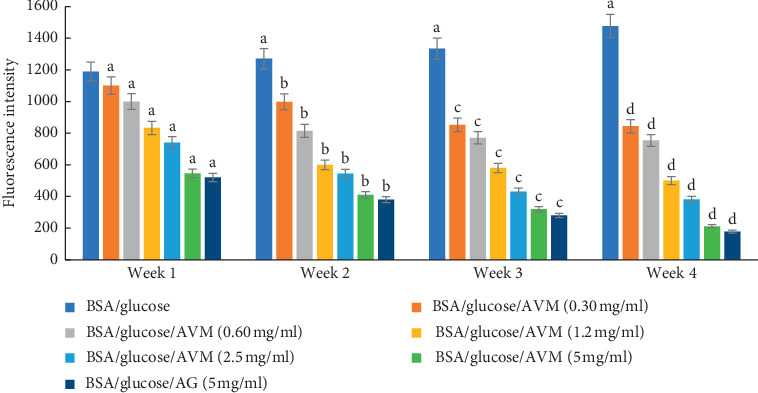
Effects of AVM extract on formation of fluorescent advanced glycation end products (AGEs) in BSA incubated with glucose. Each value represents the mean ± SE (*n* = 3). ^a^*p* < 0.05 when compared to BSA/glucose at week one; ^b^*p* < 0.05 when compared to BSA/glucose at week two; ^c^*p* < 0.05 when compared to BSA/glucose at week three; ^d^*p* < 0.05 when compared to BSA/glucose at week four.

In Figure 1, the fluorescence intensity is shown during the four weeks of incubation. The results show that fluorescence was significantly increased over the weeks in the BSA/glucose system compared to the groups incubated with BSA/glucose/AVM at different concentrations, where a significant decrease in fluorescence was observed depending on the concentration. After four weeks of the experiment, the BSA/glucose/AVM group (5 mg/ml) showed the greatest decrease in fluorescence intensity (85.64%), while the AG (5 mg/ml) showed a decrease of 87.94% in comparison with the BSA glycated in the same time interval.

#### 3.1.2. Determination of Fructosamine

The effect of AVM on fructosamine levels during the four weeks is shown in Table 1. The results show that the concentration of fructosamine in the glycosylated BSA increased significantly over time. By adding AVM to different concentrations, fructosamine levels increased at a lower rate during the four weeks of the test. In the fourth week, a fructosamine value of 77.5 mM was observed when using the BSA/glucose/AVM system (5 mg/ml), compared to the BSA/glucose/AG system, which showed a value of 75.8 mM. The glycated BSA showed a value of 119.6 mM in the same time interval.

#### 3.1.3. BSA-Methylglyoxal Assay

**Figure 2:**
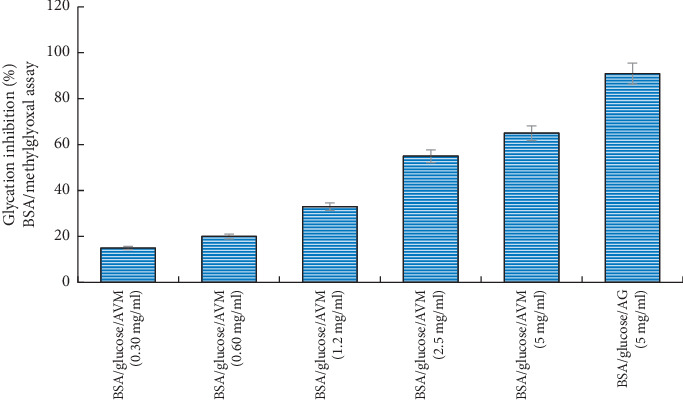
Glycation inhibitory activity analysis of the methanol extract from *Aloe vera* in BSA-methylglyoxal model. Values (mean ± standard deviation) are expressed as a percentage of glycation inhibition. AVM: methanol extract from *Aloe vera*.

The effect of AVM at different concentrations in the BSA-methylglyoxal test is shown in Figure 2. It can be observed that the greatest inhibition was presented when using the BSA/glucose/AVM system (5 mg/ml) showing an inhibition of 65%, compared to the BSA/glucose/AG system (5 mg/ml) which showed an inhibition of 91%.

#### 3.1.4. Arginine-Methylglyoxal Assay

**Figure 3:**
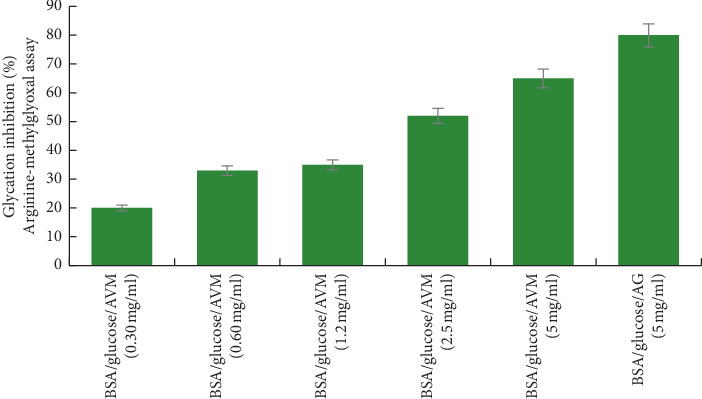
Glycation inhibitory activity analysis of the methanol extract from *Aloe vera* (AVM) in arginine-methylglyoxal model. Values (mean ± standard deviation) are expressed as a percentage of glycation inhibition. AVM: methanol extract from *Aloe vera*.

The antiglycation test using the arginine-methylglyoxal model showed that the BSA/glucose/AVM system (5 mg/ml) inhibited the generation of fluorescent AGEs at 65% (Figure 3). The BSA/glucose/AG system (5 mg/ml) showed an inhibition percentage of 80%.

#### 3.1.5. N*ε*-(Carboxymethyl) Lysine

**Figure 4:**
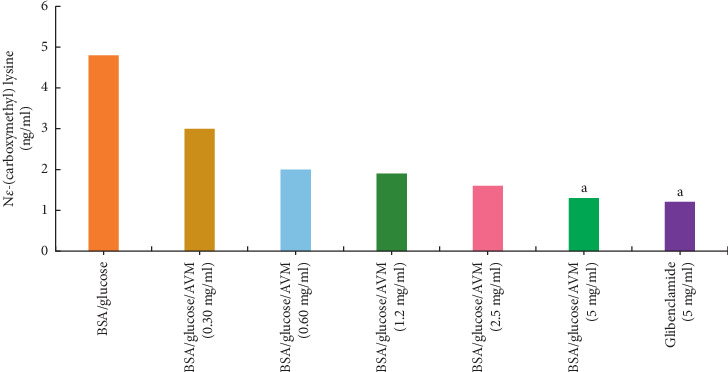
Effect of AVM on the inhibition of N*ε*-(carboxymethyl) lysine (CML) after four weeks of incubation. Each value represents the mean ± SEM (*n* = 3). ^a^*p* < 0.05 compared to BSA/glucose.

The effect of AVM on the inhibition of N*ε*-(carboxymethyl) lysine (CML) after four weeks of incubation is shown in Figure 4, where it can be seen that the concentration of CML in the BSA/glucose system increased. During the experiment, when evaluating the results of the BSA/AVM/glucose system, it was observed that the CML values were concentration-dependent, obtaining a significant 73% inhibition in the formation of CML at a concentration of 5% mg/ml, compared to the BSA/AG/glucose system (5 mg/ml), which showed a 74% inhibition compared to the glycated BSA.

### 3.2. Determination of Protein Carbonyl Content

Table 2 shows the protein carbonyl content during the four weeks. When observing the glycated BSA, an increase in protein carbonyl content can be seen during the four weeks of the experiment. While adding AVM, the BSA/glucose system at different concentrations showed a significant decrease in protein carbonyl content during the four weeks that the experiment lasted obtaining the highest decrease in the BSA/glucose/AVM system at a concentration of 5 mg/ml, which showed a value of 82.88 nmol/mg protein. The BSA/glucose/AG system and the glycated BSA showed values of 82.56 nmol/mg protein and 89.15 nmol/mg protein, respectively, in the same time interval.

### 3.3. Thiol Group Estimation

The effect of AVM during the four weeks at different concentrations is shown in Table 3. The BSA/glucose system showed a significant increase in thiol groups throughout the experiment. By adding AVM to the BSA/glucose system, a significant decrease in thiol-dependent groups can be observed. The greatest decrease was obtained at a concentration of 5 mg/ml (89.12 nmol/mg protein) at four weeks of the experiment, while the BSA/glucose/AG and glycated BSA system showed values of 78.08 and 70.05 nmol/mg protein, respectively, in the same time interval.

### 3.4. Enzymatic Assays

#### 3.4.1. *α*-Amylase, *α*-Glucosidase, and Lipase Inhibition Activity

**Figure 5:**
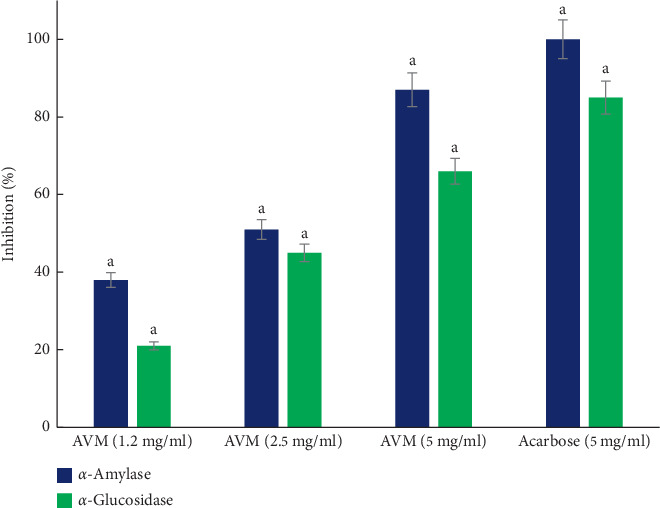
*α*-Amylase and *α*-glucosidase inhibition activity analysis of the methanol extract from *Aloe vera*. Values (mean ± standard deviation) expressed as a percentage of enzymatic inhibition. AVM: methanol extract. Letters indicate statistical significance(*p* < 0.05).

**Figure 6:**
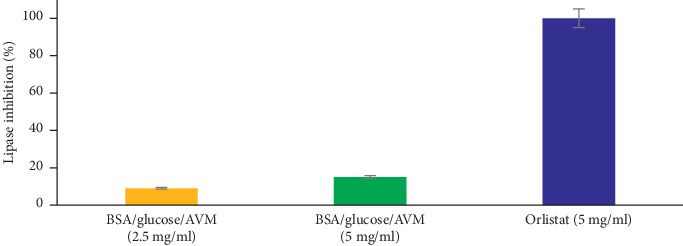
Lipase inhibition activity analysis of the methanol extract from *Aloe vera*. Values (mean ± standard deviation) expressed as a percentage of enzymatic inhibition. AVM: methanol extract.

Figure 5 shows the inhibitory effect of AVM on the activity of the alpha-amylase enzyme. The AVM concentration of 5 mg/ml showed a significant inhibition of 87%, compared to acarbose (5 mg/ml) which was used as a positive control and showed an inhibition percentage of 97%. The concentrations of 0.60 and 0.30 mg/ml AVM did not show inhibitory activity. Regarding the inhibition of the *α*-glucosidase enzyme (Figure 5), the concentration of 5 mg/ml of AVM significantly inhibited the activity of the enzyme by 66%, compared to acarbose at the same concentration, which presented an 85% inhibition. The concentrations of 0.60 and 0.30 mg/ml of the AVM extract showed no inhibition of the enzyme *α*-glucosidase. Regarding the inhibition of pancreatic lipase, the concentrations of 2.5 and 5 mg/ml of AVM showed an inhibitory activity of 9% and 15%, respectively (Figure 6), compared to the control drug, which presented an inhibition of 99%.

## 4. Discussion

AGEs are a group of compounds that are formed by the nonenzymatic reaction of reducing sugars and metabolites related to proteins and amino acids to produce Schiff bases. Subsequently, Amadori products (such as fructosamine) are formed which degrade into dicarbonyl compounds to finally produce stable AGEs [1, 12, 15]. AVM reduced fructosamine levels, which are associated with decreased AGE formation. The reduction of fructosamine, therefore, is a therapeutic way to delay incident vascular complications [16]. To this end, the capacity of AVM to inhibit the protein glycation reaction using the BSA/glucose system was evaluated. It was observed that AVM can inhibit the glycation reaction (which is related to complications in diabetes) in a way similar to the aminoguanidine (AG) used as a control. Since the formation of AGEs is favored by oxidative reactions, AVM could have inhibited them by reducing the formation of reactive oxygen species (ROS), or by eliminating the ROS formed *in vitro* by the oxidation of sugars and/or by the oxidative degradation of Amadori products [17]. It has been reported that the administration of the ethanolic extract of *A. vera* gel helps prevent excessive free radical formation through various biochemical pathways and also reduces the glycosylation of enzymes [18].

When blood sugar levels rise, the accumulation of fructose in different organs can occur. Both fructose and products derived from oxidation such as methylglyoxal can react with proteins and form AGEs [12, 15]. Arginine is one of the main targets of protein glycosylation by reactive carbonyls, which react with the guanidine group of the arginine to form AGEs [19]. Therefore, inhibiting the glycation of proteins and their oxidizing products is of paramount importance to prevent the complications of diabetes.

Our results show that the *Aloe vera* methanol extract (AVM) presents inhibitory activity in the formation of methylglyoxal and arginine, and this could be due to the presence of flavonoids [8]. It has been reported that flavonoids, as well as proanthocyanidins and phenolic acids, among others, are capable of inhibiting the glycation process [20, 21], since they inhibit the formation of AGEs by capturing precursors such as 1,2-dicarbonyl or interacting with glucose, preventing it from joining proteins.

High concentrations of postprandial glucose are a risk factor for microvascular and macrovascular complications in diabetes mellitus. AVM showed inhibitory activity of the enzymes *α*-amylase and *α*-glucosidase. Several flavonoids have been reported as inhibitors of these enzymes for their ability to bind to proteins [22], which may have contributed to the inhibitory effect of AVM. Previous studies of *Aloe vera* extract indicate that *Aloe* can act as a hypoglycemic agent through the potent inhibition of pancreatic amylase activity. This action decreases the breakdown of starch and offers good postprandial glycemic control [23].

AVM inhibited pancreatic lipase (PL), and it is known that insulin sensitivity is related to increased body fat [24], so inhibiting PL can be a way to inhibit fat absorption. Previous studies report that flavonoids inhibit PL, since these compounds can inactivate lipase through nonspecific binding of the enzyme, which could explain the inhibitory activity of AVM.

It has been reported that AG inhibits protein glycosylation, eliminating beta-dicarbonyls [2]. However, in clinical trials, it showed adverse effects such as gastrointestinal disorders, vasculitis, and abnormal liver function, while, in diabetic rats, it showed renal tumors [25].

Therefore, the discovery of inhibitors of the protein glycation reaction to reduce the formation of AGEs provides a promising therapeutic approach to prevent and treat diabetic complications.

Carboxymethyl lysine (CML) is formed by the oxidative degradation of Amadori products [19], and it is one of the best characterized AGEs chemically, which has been used as the main marker of AGEs in many studies [26]. Recent research has shown that CML can interact with the RAGE receptor by inactivating signaling pathways, which leads to the expression of proinflammatory genes [27]. CML has been found in the blood vessels of the retina in people with type 2 diabetes mellitus, and it has been linked to the degree of retinopathy [28], and therefore, it is important to find compounds that inhibit the formation of CML. In this regard, AVM showed the ability to reduce the formation of CML significantly, which could be an alternative to reduce the complications of diabetes associated with AGEs.

Protein carbonyl compounds are formed by the oxidation of amino acid residues, which, together with the protein thiol groups, are considered as markers of the modification of proteins by oxidation. This modification is measured by the formation of hydrazone 2,4-dinitrophenylhydrazine adducts (DNPH) [2]. In this study, it was observed that AVM is effective in eliminating carbonyls bound to proteins and increasing the concentration of thiol protein groups, helping to prevent oxidative damage in proteins.

## 5. Conclusions

*Aloe vera* methanol extract effectively inhibited the glycation reaction of proteins in the BSA/glucose system, possibly due to the oxidative degradation of fructosamine; however, it is necessary to determine the mechanism of action by which AVM inhibits AGEs. More research is required to describe the interactions of AVM with the enzymes *α*-amylase, *α*-glucosidase, and pancreatic lipase to provide a basis for the development of new natural inhibitors.

## Figures and Tables

**Table 1:** Effect of AVM on fructosamine levels during four weeks of incubation using the BSA/glucose system.

Experimental groups	Fructosamine (mM)			
	Week 1	Week 2	Week 3	Week 4
BSA/glucose	87.2 ± 0.8	97.9 ± 0.9	108.5 ± 1.6	119.6 ± 1.2
BSA/glucose/AVM (0.30 mg/ml)	86.7 ± 1.2	85.5 ± 1.3^a^	86.3 ± 0.8^a^	86.9 ± 1.4^a^
BSA/glucose/AVM (0.60 mg/ml)	83.4 ± 0.8^a^	84.3 ± 0.5^b^	85.1 ± 1.2^c^	85.6 ± 1.7^d^
BSA/glucose/AVM (1.2 mg/ml)	80.1 ± 1.1^a^	82.7 ± 1.3^b^	83.5 ± 0.9^c^	84.1 ± 1.3^d^
BSA/glucose/AVM (2.5 mg/ml)	77.8 ± 1.2^a^	79.2 ± 1.8^b^	81.4 ± 0.5^c^	82.7 ± 1.4^d^
BSA/glucose/AVM (5 mg/ml)	74.4 ± 1.5^a^	75.4 ± 1.2^b^	77.1 ± 0.6^c^	77.5 ± 1.4^d^
BSA/glucose/AG (5 mg/ml)	72.1 ± 1.4^a^	73.9 ± 1.5^b^	74.5 ± 1.2^c^	75.8 ± 0.9^d^

Results are expressed as mean ± SEM (*n* = 3). ^a^*p* < 0.05 when compared to BSA/glucose at week one; ^b^*p* < 0.05 when compared to BSA/glucose at week two; ^c^*p* < 0.05 when compared to BSA/glucose at week three; ^d^*p* < 0.05 when compared to BSA/fructose at week four.

**Table 2:** Effect of AVM on protein carbonyl content using the BSA/glucose system.

Experimental groups	Protein carbonyl content (nmol/mg protein)			
	Week 1	Week 2	Week 3	Week 4
BSA/glucose	87.72 ± 1.8	88.59 ± 1.4^a^	88.91 ± 1.4^a^	89.15 ± 1.2^a^
BSA/glucose/AVM (0.30 mg/ml)	86.44 ± 0.7^a^	87.19 ± 0.8^b^	87.09 ± 1.1^c^	86.92 ± 1.1^d^
BSA/glucose/AVM (0.60 mg/ml)	86.31 ± 1.1^a^	87.04 ± 1.7^b^	86.53 ± 1.4^c^	86.37 ± 1.1^d^
BSA/glucose/AVM (1.2 mg/ml)	85.11 ± 9.9^a^	86.17 ± 1.4^b^	86.81 ± 1.2^c^	85.07 ± 0.9^d^
BSA/glucose/AVM (2.5 mg/ml)	84.33 ± 1.2^a^	85.94 ± 0.8^b^	84.18 ± 1.4^c^	84.66 ± 1.1^d^
BSA/glucose/AVM (5 mg/ml)	83.24 ± 0.6^a^	84.33 ± 0.7^b^	83.57 ± 0.7^c^	82.88 ± 0.6^d^
BSA/glucose/AG (5 mg/ml)	83.41 ± 1.7^a^	83.78 ± 0.7^b^	82.92 ± 0.3^c^	82.56 ± 1.1^d^

Results are expressed as mean ± SEM (*n* = 3). ^a^*p* < 0.05 when compared to BSA/fructose at week one; ^b^*p* < 0.05 when compared to BSA/fructose at week two; ^c^*p* < 0.05 when compared to BSA/fructose at week three; ^d^*p* < 0.05 when compared to BSA/fructose at week four.

**Table 3:** Effect of AVM on thiol group using the BSA/glucose system.

Experimental groups	Thiol group (nmol/mg protein)			
	Week 1	Week 2	Week 3	Week 4
BSA/glucose	77.08 ± 1.2	75.38 ± 0.07^a^	75.88 ± 0.05^a^	70.05 ± 0.08^a^
BSA/glucose/AVM (0.30 mg/ml)	75.95 ± 1.0	77.73 ± 0.09^b^	79.16 ± 0.07^b^	79.52 ± 0.06
BSA/glucose/AVM (0.60 mg/ml)	77.08 ± 0.51	78.85 ± 0.12^b^	79.31 ± 0.08^b^	84.43 ± 0.04^d^
BSA/glucose/AVM (1.2 mg/ml)	76.29 ± 0.83^a^	79.79 ± 0.09^b^	80.46 ± 0.11^b^	86.60 ± 0.09^d^
BSA/glucose/AVM (2.5 mg/ml)	76.41 ± 0.11^a^	81.38 ± 0.07^b^	83.68 ± 0.08^b^	87.06 ± 0.04^d^
BSA/glucose/AVM (5 mg/ml)	76.68 ± 0.08^a^	83.29 ± 0.05^b^	85.61 ± 0.09^b^	89.12 ± 0.06^d^
BSA/glucose/AG (5 mg/ml)	76.52 ± 0.08^a^	77.37 ± 0.02^b^	77.56 ± 0.04^b^	78.08 ± 0.09^d^

Results are expressed as mean ± SEM (*n* = 3). ^a^*p* < 0.05 when compared to BSA/fructose at week one; ^b^*p* < 0.05 when compared to BSA/fructose at week two; ^c^*p* < 0.05 when compared to BSA/fructose at week three; ^d^*p* < 0.05 when compared to BSA/fructose at week four.

## Data Availability

The data used to support the findings of this study are included within the article.
